# Human-Inspired Online Path Planning and Biped Walking Realization in Unknown Environment

**DOI:** 10.3389/fnbot.2019.00036

**Published:** 2019-06-04

**Authors:** Mirko Raković, Srdjan Savić, José Santos-Victor, Milutin Nikolić, Branislav Borovac

**Affiliations:** ^1^Faculty of Technical Sciences, University of Novi Sad, Novi Sad, Serbia; ^2^Institute for Systems and Robotics, Instituto Superior Tecnico, University of Lisbon, Lisbon, Portugal

**Keywords:** humanoid robot, bipedal locomotion, motion primitives, path planning, clothoid, walk realization

## Abstract

The focus of research in biped locomotion has moved toward real-life scenario applications, like walking on uneven terrain, passing through doors, climbing stairs and ladders. As a result, we are witnessing significant advances in the locomotion of biped robots, enabling them to move in hazardous environments while simultaneously accomplishing complex manipulation tasks. Yet, considering walking in an unknown environment, the efficiency of humanoid robots is still far from being comparable with the human. Currently, bipeds are very sensitive to external changes and they have severe constraints for adaptation of walk to conditions from such a complex environment. Promising approaches for efficient generation and realization of walking in a complex environment are based on biological solutions that have been developed for many years of evolution. This work presents one such human-inspired methodology for path planning and realization of biped walk appropriate for motion in a complex unfamiliar environment. Path planning results in calculating clothoid curves that represent well the human-like walking path. The robot walk is realized by the composition of parametric motion primitives. Such an approach enables on-line modification of planned path and walk parameters at any moment, instantly. To establish the relationship between high-level path planner and the low-level joint motion realization, we had to find a way to extract the parameters of the clothoid paths that can be linked with the parameters of the walk and consequently to motion primitive parameters. This enabled the robot to adopt its walking for avoiding the obstacles and for a smooth transition between different paths. In this paper we provide a complete framework that integrates the following components: (i) bio-inspired online path planning, (ii) path-dependent automatic calculation of high-level gait parameters (step length, walking speed, direction, and the height of the foot sole), and (iii) automatic calculation of low-level joint movements and corresponding control terms (driving motor voltage) through the adaptation of motion primitives which realize walking pattern and preserves the dynamic balance of the robot.

## 1. Introduction

We are witnessing that jobs which were in the past exclusively handled by humans, spanning from the care of patients in hospitals to playing musical instruments, are now possible to be carried out by robots. One of the reasons for this achievement lies in the fact that biomechanics of human activity (including walk) has been deeply studied and explored (Winter, 2009; Lin and Pandy, 2017; Young et al., 2017). The processing power of the computer has increased to the extent where it is possible to adopt a complex control algorithm for the real-time whole body control of the robot with numerous DOFs (Degrees Of Freedom) (Moro and Sentis, 2019). The focus of biped locomotion research has moved toward real-life scenario applications, like walking on uneven terrain, passing through doors, climbing stairs, and ladders, etc. This is mainly motivated by the Fukushima disaster and the DARPA Robotics Challenge (Krotkov et al., 2017). As a result, numerous robots have been rapidly developed to become machines that can move in unknown environments, simultaneously accomplishing complex tasks (Schwarz et al., 2016; Tsagarakis et al., 2017; Asfour et al., 2019).

Humanoid robot walk is usually evaluated against human walking performance. However, humans develop the walking skills since the very first year of their life, with constant extensive improvements and adaptation to the changes in the kinematics and dynamics of their body. Walking, as time goes by, becomes so perfected and automated that we are not aware of the complexity of the underlying sensory-motor control system. Most humans can seamlessly perform various types of walk, compensate for external disturbances, adapt to different complex ground structures, and simultaneously perform other tasks while communicating with others, manipulating objects etc. In combination with perception (mainly vision), humans can easily cope with an unknown surrounding, and reach their goal while avoiding and/or overcoming various obstacles, stairs, doors, and other objects commonly found in the human environment (Smulders et al., 2012).

Development of humanoids with such skills is obviously a challenging task. Humanoids should be able to plan the path online and adapt the walk realization in order to follow the path close enough. This task is accompanied by the need to constantly prevent the robot from falling, i.e., to preserve its dynamic balance. The well-known concept of Zero Moment Point (ZMP) introduced by Vukobratović and Borovac (2004), defines the conditions to preserve a dynamic balance of bipeds (Vukobratovic et al., 2012). One of the most recurring ZMP based approaches for walk synthesis is ZMP preview controller introduced by Kajita et al. (2003). In this method used by Morisawa et al. (2007) and Perrin et al. (2012) among others, the robot needs to predefine the position of footprints, calculate the trajectory of the ZMP, the motion of the center of mass and finally the motion of the rest of the system.

However, humans do not plan and define the footprints on the ground before they start to walk. If it is expected from the robots to operate in the human everyday environment, the walking skills of the robot have to be as close as possible to those of humans. The possibility to adapt to the current state of the environment (to modify the online walking path and adapt gait parameters) is a necessity. Also, due to the always present disturbances, the control system has to be robust, fast, and responsive in order to successfully compensate for them. In addition, path generation (approaching, avoiding, and going around obstacles) should be inspired by the way humans perform this. Thus, the system for motion synthesis has to offer a biologically inspired online modification of planned path and walking parameters and be supported by a robust motion control system that can compensate disturbances while constantly preserving dynamic balance. Path planner introduced by Dornbush et al. (2018) decomposes the task into a sequence of smaller tasks and focuses the planning efforts to reason over much smaller search spaces. We find this is a human-like approach were local obstacles should be treated first, but always working on solving the main task. Kumagai et al. (2018) proposed an efficient footstep planning for the robot to traverse an unknown narrow space in a human-like manner. Although the robot is demonstrating the ability to move in highly cluttered space, the algorithm that does not require the footstep planning should be considered.

Taking into consideration adaptability, responsiveness, and robustness without the need for precise trajectory execution, a methodology that enables synthesis of online modifiable walk based on reconfigurable adaptive motion primitives (RAMPs) is introduced in Raković et al. (2014). The RAMP is a parametric movement with the relationship established between its parameters and the walking characteristics. We find that this approach offers an intuitive interface for integration with higher level path planning algorithms. Thus, any requirement for modification of characteristics of walking (for example, speed, stride, direction etc.) will instantly influence the change in RAMPs and as a result, a modified gait will emerge. RAMPs are inspired by neurological studies (Giszter et al., 1993) that showed the synergistic motion of leg joints caused by electrical microstimulation. The experiment showed that the same stimulation drives the leg toward the equilibrium point irrespective of the initial position. The motion of the foot corresponds to a vector field that was convergent toward an equilibrium point characterized by the location and intensity of stimulation (Mussa-Ivaldi et al., 1994). The same approach is embedded in the properties of RAMPs.

The use of primitive movements as building blocks for generating more complex movements is not a unique approach. In the last decade, several motion primitive methodologies have been defined. Hauser et al. (2008) introduced a library of steps, where each different step is one primitive. Zhang et al. (2008) used recordings of the human to segment the movement of the leg into motion primitives. Moro et al. (2014) introduced kinematic movement primitives derived with principal component analysis from recorded human movements that can be combined to define a complex motion applicable for the realization of the robot walking. Ijspeert et al. (2002) and Schaal (2006) defined most known motion primitive methodology called Dynamic Movement Primitives. It models the robotic joint or end-effector trajectories as a combination of nonlinear dynamical systems. Statistical learning techniques are used to learn how to combine these primitive movements to code basic rhythmic or discrete behavioral patterns. In Gams et al. (2014), extended Coupling Movement Primitives are presented to model the interaction of robot with objects and humans.

In this paper, the RAMPs methodology for walk realization is augmented with the biologically inspired on-line path planning algorithm. The path planning algorithm is developed, that is inspired by the behavior of humans walking in an unknown environment. It enables the robot to avoid obstacles in a human-like manned in a cluttered environment. Simulation results show that the biped can adapt its walking path and gait parameters online. The path can be re-planned when needed, depending on the localization of the objects, i.e., obstacles on the robot's way toward the goal position. The proposed approach searches for the transitory goal position in close robot's surrounding and generates a path that is far enough from the obstacles. The parameters of the path are linked with the parameters of the walk (speed, direction, step length) that are thus driving the robot to adapt to a new path geometry.

The contribution of this work is the framework for online human-like path planning and walking that integrates (i) the human-inspired path planning algorithm, (ii) the link between the parameters of the walk and the RAMP parameters for defining joints motion, and (iii) the dynamic balance controller that compensates for disturbances and ensures the dynamic balance of the robot. The developed planning and control ensures that the robot follows the online generated walking path and preserves its balance. The automatic calculation of the walking parameters with respect to the geometry and current position of the robot is proposed. In section 2.1 the motivation and a brief overview of the methodology for generating an online modifiable walk based on primitives are presented. Afterward, in section 2.2, the algorithm for the path generation and calculation of walk parameters are introduced, followed by the description of the algorithm for path planning, given in section 2.3. To show the abilities of the framework, section 3 presents the simulation results of the biped walking in the environment with different obstacles. The simulation involves a complete dynamic robot model with many degrees of freedom. The results show that the robot can realize dynamically balanced walk while on-line changing the path and modifying the walking parameters. Examples show hot the robot avoids static and moving obstacles in order to reach the goal position while following the human-like shape of the walking path. The paper concludes and proposes the direction for further research in section 4.

## 2. Motivation, Approach, and Methods

### 2.1. Motivation for Composing the Walk From Motion Primitives

Let us suppose that the robot has the task to go to the other part of the unknown room to pick an object. The task a robot needs to solve is similar to what the human is solving. The human will scan for the obstacles and make a plan for avoiding one obstacle at a time (Erni and Dietz, 2001). The common approach for solving this task in humanoid robotics is based on the use of well-known planners to generate the safe path toward the goal (Kuindersma et al., 2016), usually followed by the foot placement algorithm and precise realization of generated trajectories. Other approaches, such as Zaytsev et al. (2015), propose that the planner should focus on local tasks, and plan only a few steps ahead. This approach is more biologically inspired since it does not require precise execution of the preplanned foot placements and precise realization of calculated joint trajectories.

Reconfigurable Adaptive Motion Primitives is the methodology that enables the realization of complex motion such as walking that does not require the programming of joint trajectories for a full step in advance. The realization of motion is defined with the set of parameters that are defining the goal position of the end of the kinematic chain with respect to the base coordinate frame of the robot. The result is the simultaneous motion of joints that are driving the end-effector (foot, head, or hand) toward parametric equilibrium point. These parameters can be changed at any time instant. In Borovac and Raković (2011), different methods based on model learning and inverse kinematics are presented.

**Figure 1:**
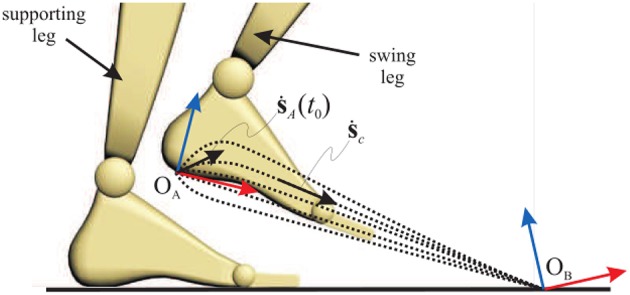
Continuation of leg stretching after bending is completed with possible trajectories of the foot that depends on the initial velocity at the moment when stretching starts.

The shape of the motion of the kinematic chain is smooth, including migration between the execution of the consequent primitives. The smoothness is achieved by a gradual change in the velocities of end-effectors toward the new equilibrium. An example of a primitive that is a part of a walking cycle is leg stretching (Figure 1) that incorporates the simultaneous motion of leg joints. It is followed by the completion of leg bending, with the smooth transition of the foot velocity toward the new end goal position.

The velocity of foot ${\dot{\text{s}}}_{A}$ is determined from the following equation:

(1) $${\dot{s}}_{A}\left(t_{i}\right)=\left(1-b\left(t_{i}\right)\right).\;{\dot{s}}_{A}^{0}+b\left(t_{i}\right).\left[\begin{array}{c} v_{int}\;.\;p_{e}^{ort}\\ \omega_{int}\;.\;o_{e}^{ort} \end{array}\right]$$

where ${\text{p}}_{e}^{ort}$ and ${\text{o}}_{e}^{ort}$ are the unit vectors of the error of position and orientation of the foot with respect the end-goal position. The coefficient *b* ensures the smooth transition in intensity and direction of the heel velocity.

**Figure 2:**
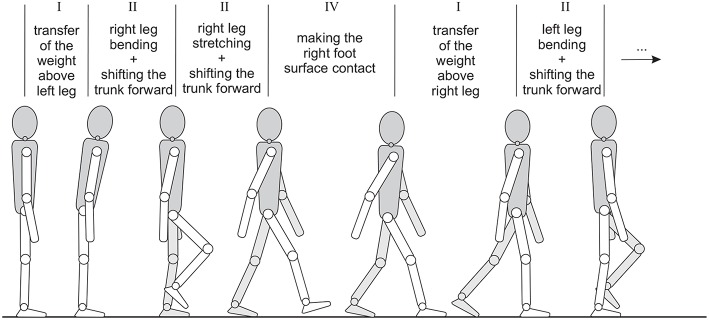
Decomposition of the walk into the phases that can be realized by motion primitives (Raković et al., 2014).

The basic walk (i.e., walking on a flat surface) can be decomposed into simple movements, similar to the previously described leg stretching (Figure 2).

Altogether five different primitives have been identified for leg movement, as well as one for the trunk and one for the arms. The legs can perform: (i) leg bending, (ii) leg stretching, (iii) inclining the robot forward during single support phase, (iv) making the foot surface contact after the heel strike, and (v) transferring the body weight onto the subsequent supporting leg during double support phase. Two additional primitives are the (vi) primitive for maintaining the trunk in the upright posture and the (vii) arm swing primitive, that represents natural motion opposing the leg motion. Arm swinging reduces the angular momentum of the body, compensates the rotational motion caused by the legs and contributes to the preservation of dynamic balance.

**Figure 3:**
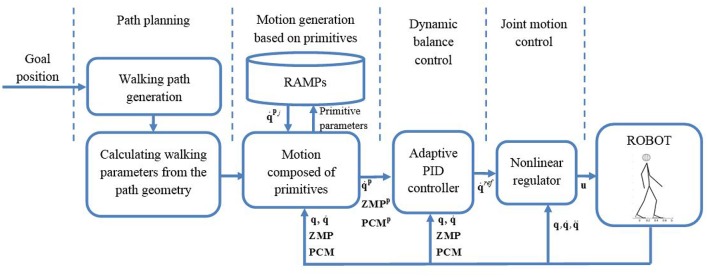
Cascade walking controller composed of the modules for path planning, motion generation with RAMPs, dynamic balance preservation, and joint motion control.

A critical segment in walk realization is the preservation of dynamic balance. Each motion primitive ensures an appropriate shape of the end-effector trajectories and smooth transition from the previous primitive, but it does not ensure the preservation of dynamic balance. To fulfill these requirements, from path planning to execution of motion on a joint level, we adopted a cascade controller (Figure 3) that consists of four blocks: (i) block for path planning, (ii) block for tying motion primitives, (iii) dynamic balance controller, and (iv) joint motion controller.

The input to the control system is a goal position that the robot should reach. The first block calculates the path toward the goal or transitory position in case of obstacle avoidance. The second block determines the desired joint angular velocities based on a set of tied-up primitives. Since motion primitives do not consider the dynamic balance of the system, the third block is introducing the corrections of desired joint velocities taking into consideration current position of ZMP and projection of the center of mass to ensure the preservation of dynamic balance. Finally, the fourth block is calculating the joint control values. Since the humanoid robot is highly non-linear and highly coupled system, the fourth block is composed of feedback linearization, sliding mode control and disturbance estimator.

**Figure 4:**
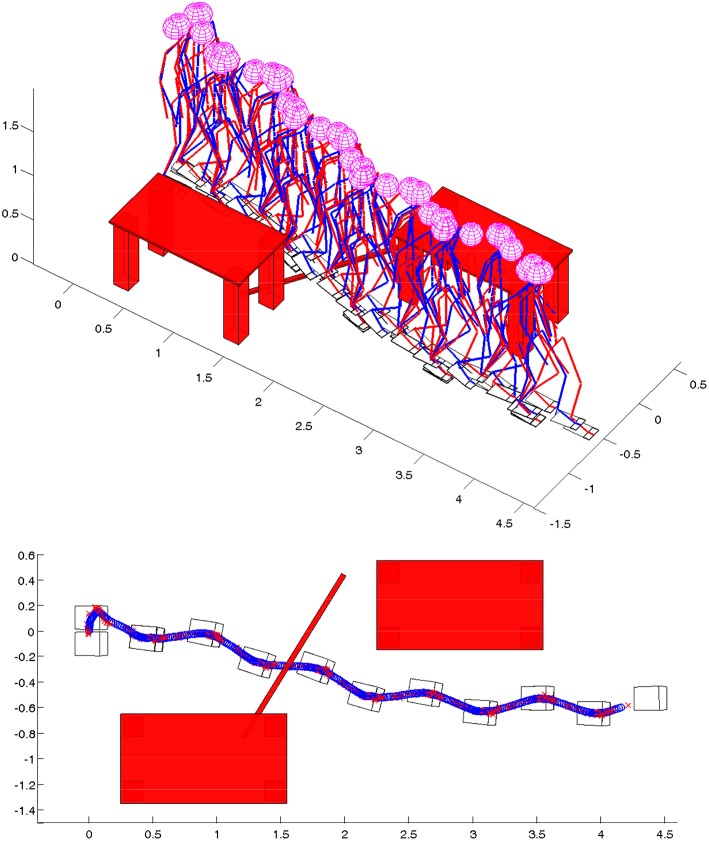
Stick diagram, footprints, and trajectory of ZMP (red cross) and PCM (blue circle) for on-line adaptation of walking direction and speed while avoiding obstacles (Raković et al., 2014).

Walk synthesized in this way can be changed online, meaning that overall motion parameters are introduced: walk speed *W*_*Speed*_, the height of the leg during the swing phase *W*_*Height*_, step length *W*_*Length*_ and walking direction *W*_*D*_*ir*. The relationship between these parameters and parameters of the primitives is established which causes an immediate and automatic change of the parameters of the primitives with the change in the parameters of the walk. An example of the simulated biped walk where the robot is changing the parameters of the walk online, in order to pass between the tables and step over the bar on the ground, is shown in Figure 4.

### 2.2. Path Generation With Clothoid Curves

The automatic calculation of four walking parameters (*W*_*Speed*_, *W*_*D*_*ir*, *W*_*Height*_, and *W*_*Length*_) can be determined from the geometry of the path. Thus, an approach for generating the path, and for the calculation of the walking parameters based on its geometry is introduced. Several problems were addressed in our analysis. An important question is whether the four walking parameters are independent or is there some relationship established among them. Another important question concerned the shape of the human walking path. Some insights from physiology and biomechanics were used in our research to tackle these problems and to achieve biologically inspired, anthropomorphic robot walk. In Arechavaleta et al. (2008), possible strategies were investigated, focusing on the formation of a walking path with given goal position. The assumption was that the path is chosen according to some optimization principle. The results in Arechavaleta et al. (2008), have shown that the cost function to be minimized is a variation (time derivative) of the path curvature which implies that clothoid arcs are a good approximation of a walking path. This conclusion was adopted and clothoid arcs have been chosen for path shape in our path planning algorithm.

Having defined the coordinates of initial (i.e., current) position of the robot and the desired goal position^1^ of the robot, a G1 fitting (Bertolazzi and Frego, 2015) with clothoid curve can be used to fit between these two coordinates. This way, it is possible to set a new goal point at any time instant and to fit a new clothoid arc between the current position and the new goal position. This enables online modification of a walking path in case of unpredicted or dynamic obstacles while ensuring the smooth path transition.

**Figure 5:**
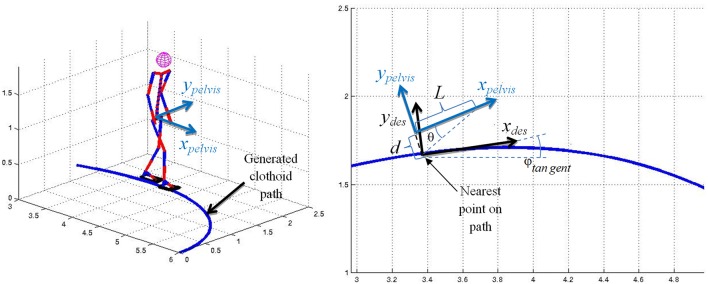
Calculation of *W*_*Dir*_ according to the path geometry.

In this paper, forward goal-oriented locomotion without backward and side steps has been considered. In this case, robot locomotion can be simply approximated with a nonholonomic unicycle model which implies the dependency between the walking direction and the tangent to the walking trajectory. This dependency is used to calculate one of the walk parameters, i.e., walking direction. In each time instance, a minimal distance is calculated from the clothoid curve to the coordinate system attached to the robot pelvis. When the point, with the minimal distance from the robot base, is determined, the tangent on the curve in that point is calculated. The idea is to maintain the current orientation of the *x*-axis of the robot base frame (*x*_*pelvis*_) and the *x*-axis of the frame in the nearest point (*x*_*des*_), determined by the path tangent in that point (Figure 5).

The angle between the curve tangent (*x*_*des*_) and *x*_0_ axis of the global coordinate frame, is denoted as *φ*_*tangent*_. If we imagine that the robot started with some initial offset from the desired path, the robot would walk parallel to the path, keeping the initial offset. Therefore, it is necessary to include some corrective, feedback term, which would return the robot on the desired path if it gets off the track. This feedback term is given with the second term in the equation:

(2) $$\begin{array}{r} W_{Dir}=\varphi_{tangent}+K_{P}\cdot arctg\left(\frac{d}{l}\right) \end{array}$$

where *φ*_*tangent*_ is the angle of the path tangent, *K*_*P*_ is proportional gain, *d* is the distance from the path and *l* is the step length.

When the robot base frame crosses the path curve, the sign of the proportional gain *K*_*P*_ changes. This sign is determined on the basis of the sign of the cross product of the path tangent vector, in the point with minimal distance from the robot, and the vector pointing from the robot base frame toward the nearest point on the path. This feedback term tends to minimize the distance between the path and the robot base frame and it has only a proportional gain *K*_*P*_ which depends on the distance from the current to the goal position. If the robot is far from the goal this gain is smaller, and the robot gradually compensates the offset from the desired path. If the robot is close to the goal this gain is higher and the robot acts more rapidly in order to get on the desired course on time. This simple controller actually behaves like a bang-bang controller and causes the robot to walk zig-zag around the desired path. Of course, this zig-zag motion is very small, cannot be noticed and does not disturb the dynamic balance. To the human eye, the robot appears to follow the desired path accurately.

Recent studies in the field of gait physiology and biomechanics (Egerton et al., 2011) confirms that there exists a relationship between stride length and cadence which contributes to the automatic gait control mechanism. Stride length is defined as the distance between one heel strike to the next of the same foot in the walking plane. It has been proven that this relationship is linear when the subjects are walking at the self-selected speed. So the faster walking implies longer steps to a certain boundary, which is called the breakpoint. Breakpoint happens at cadences greater than 150 steps/min. At this point, stride length starts to decrease with a quadratic relationship and a further increase in cadence. This is an extreme case which is not considered in this paper. Thus, the linear relationship (*y* = *b*_1_ · *x* + *b*_0_) between stride length and cadence, with the slope *b*_1_ = 0.01 and the intercept *b*_0_ = 0.54 is adopted. Since walking speed is the product of cadence and stride length, the relationship between two walking parameters, *W*_*speed*_ and *W*_*Length*_, is provided. It is sufficient therefore to define just one of them while the other can be calculated.

Another result provides the relationship between the path curvature and walking speed. This relation, known as the 1/3 power law, is given with the following equation:

(3) $$\begin{array}{r} W_{Speed}=K\cdot R^{\beta } \end{array}$$

where *K* is the velocity gain factor, *R* is the radius of the curve and *β* is the power (having the value of 1/3). Velocity gain factor *K* is calculated as $W_{Speed}^{Des}\cdot {\left(1/2\right)}^{\beta }$, where $W_{Speed}^{Des}$ is speed of straight walking. In this case, if the radius of the curve is 2 m, the walking speed is equal to $W_{Speed}^{Des}$, if the radius is >2 m, the speed will increase, and if it is <2 m the speed will decrease. Additionally, the minimum and maximum speeds are introduced as $0.5\cdot W_{Speed}^{Des}$ and $2\cdot W_{Speed}^{Des}$ respectively.

Although this relationship was considered to be valid for a long period of time, recent studies (Olivier and Cretual, 2007) have shown that this coupling between velocity and curvature is not general. In Olivier and Cretual (2007), authors studied the velocity/curvature relationship during a single turning task. Their results have shown that power law does not apply to this situation where subjects are free to choose a walking trajectory. However, power law has been proven to be valid for predefined paths and long-term control of the turning task. Also, it was shown that the value of the power is not always 1/3 and that it depends on the geometry of the path. Since the path in our algorithm is predefined, in the form of the clothoid, and there is no final, unambiguous conclusion on this issue—the 1/3 power law has been adopted as the relationship between path curvature and walking speed. The fourth walking parameter *W*_*H*_*eight* is independent of other parameters and depends only on the estimated value of the obstacle's height if there is an obstacle to be stepped over.

**Figure 6:**
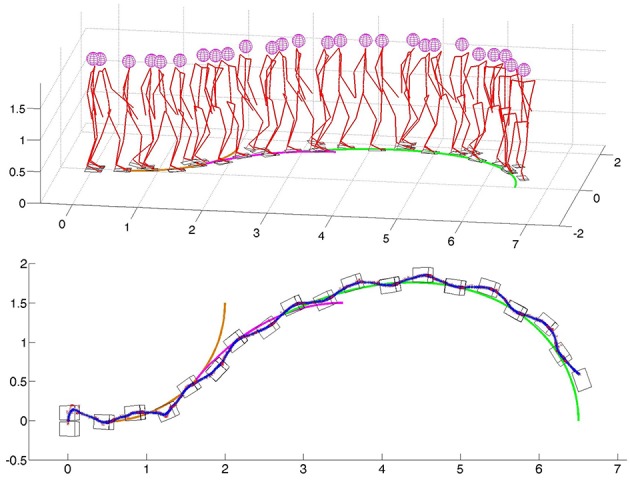
Online modification of walking parameters for walking on a path consisted of three clothoid curves: **(top)** stick diagram for the robot, **(bottom)** path, footprints, ZMP, and PCM trajectories.

In Figure 6, the robot is walking on a path that is generated according to a previously described procedure. The path is generated first to reach the goal position with coordinates *x* = 1.5 m, *y* = 2 m, and *φ* = *π*/2 (orange line). In randomly selected time instants the path is re-planned with the goal position *x* = 3.5 m, *y* = 1.5 m, and *φ* = 0 (purple line). Again, nearly before reaching the second goal position, the third goal position *x* = 6.5 m, *y* = 0 m, and *φ* = −*π*/2 is selected (green line) and a path is generated for the third time. For all three paths, as a starting point either the nearest point on the existing path is selected, or the current position of the pelvis in the case when the path is not generated. Figure 6 shows the robot's online change in walking direction, as well as in walking speed and step length to comply with the generated paths.

### 2.3. Online Path Planning Algorithm

**Figure 7:**
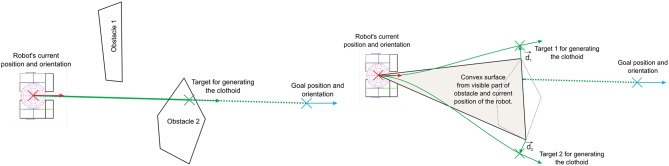
Illustration of the path that requires to be re-planed to avoid the obstacle **(left)** and illustration of the path finding for the obstacle avoidance **(right)**.

The inputs for generating the clothoid curves are the current position of the robot, given with coordinates *x*, *y*, and *φ*, and the desired goal position given with coordinates *x*_*des*_, *y*_*des*_, and *φ*_*des*_. If there is an obstacle between the current and the goal position [see Figure 7 (left)], an algorithm for finding via points for avoiding obstacles is needed. In this section, an approach is described, for determining the coordinates of the intermediate desired goal positions for generating the clothoid curves, in order to avoid the obstacles and to reach the final goal position.

The algorithm first tries to generate the shortest clothoid between current and goal position. When the clothoid is generated it is checked whether the path will ensure minimum clearance between the robot and the obstacles. The clearance value takes into account the minimum desired margin between the robot and the obstacle^2^. If the clearance is satisfied, the clothoid is generated, and the robot starts to follow the path. If not, the procedure for finding two intermediate targets is executed. These intermediate targets provide two possible paths for bypassing the obstacle. This procedure takes into account, the current position of the robot and visible part of the obstacles [see Figure 7 (right)]. These positions are used to calculate the convex surface. As a result, three points (current robot position and two points on the obstacle) constitute the corners of the convex surface.

Current position of the robot and two points are used to calculate vectors $\vec{d_{1}}$ and $\vec{d_{2}}$. The vectors $\vec{d_{1}}$ and $\vec{d_{2}}$ are the unit vectors perpendicular to the line that connects the current robot's position and two possible target positions [Target 1 and Target 2 in Figure 7 (right)]. Initial values of two target positions are actually two points on the obstacle. The target positions are iteratively modified by shifting them in the direction determined by the corresponding unit vectors $\vec{d_{1}}$ and $\vec{d_{2}}$. The modified target positions are used to generate two clothoid curves for which it is checked if they are satisfying the clearance from the obstacles. If there is sufficient clearance the clothoid is generated and the procedure is stopped. The second condition for stopping the procedure is that the orientation of the target positions is more than 180^*o*^ from the current orientation, which means the target points are on the back side of the robot. If this situation for both points occurs, the robot is surrounded by obstacles and it is not possible to find intermediate points to calculate the clothoid path.

Out of the first clothoid curve that has a clearance of two possible intermediate goal positions, the one that is closer to the robot's final position is selected. This algorithm is executed for every obstacle in the scene that is in the visible range^3^. Generated clothoid path to the goal target has to fulfill the minimum clearance constraint for each obstacle. In the case of multiple possible targets, the one with a minimal distance from the robot's current position is chosen.

**Figure 8:**
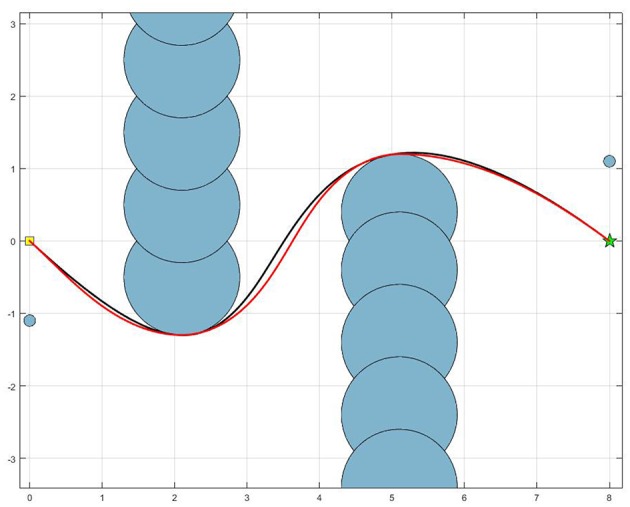
Comparison of the paths generated using PSO (black line) and using clothoids (red line). Blue circles represent static obstacles.

Our path planning algorithm, based on clothoids, has been compared with another path planning algorithm, based on particle swarm optimization (Poli et al., 2007; YarpizPSO, 2019). Particle Swarm Optimization (PSO) has been reported in literature many times for robot path planning in dynamic environment (Raja and Pugazhenthi, 2012). However, our focus is on the comparison of geometrical properties of the paths, generated by the two aforementioned approaches. Therefore, we considered that, without loss of generality, static environment with static obstacles is sufficient for such comparison. Figure 8 shows the comparison of the two paths, generated between the starting position (yellow square) and the goal position (green star), avoiding a set of static obstacles (blue circles).

**Figure 9:**
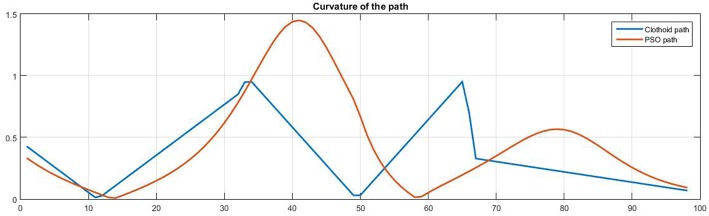
Curvature of the paths generated using PSO (red line) and using clothoids (blue line).

To geometrically compare the two paths, a curvature of each path has been calculated as the inverse of the path radius at each point. The comparison of the two curvatures is shown in Figure 9. It can be seen that the clothoid path, generated with our approach, has smaller curvature values, compared to the path generated by the PSO-based approach. According to the Equation (3), this implies that our approach, due to a smaller curvature, may provide higher walking speeds, then the approach based on PSO. However, it should be noticed that the clothoid path has the sharper changes of the curvature, meaning that its derivative would have higher peaks, compared to the PSO-based path. It means that PSO algorithm provides smoother changes in the curvature, but at the cost of higher values of this curvature, leading to the less intensive change in the direction of the walk, but limiting the walking speed, while our approach does the opposite.

The time to generate path using PSO took 13.3349 s, whereas the time to generate the path using clothoids took 0.0233 s [on Intel(R) Quad Core(TM) i5-4590 CPU at 3.30 GHz]. This significant difference emphasizes the main contribution of our approach and qualifies clothoid path planning algorithm as suitable for online path planning. We have shown that our path planning approach is computationally less expensive then the approach based on particle swarm optimization, while providing the minimum curvature of the generated path.

## 3. Simulation Model and Results

**Figure 10:**
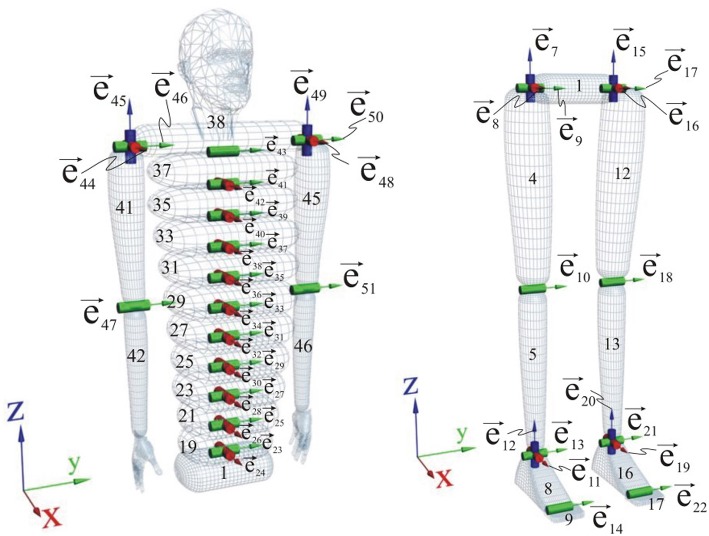
Kinematic structure of the robot model: **(left)** the trunk and the arms; **(right)** the legs.

The concept of a free-flying mechanism has been used for robot modeling. The model can have multiple kinematic chains, composed of links with one rotational degree of freedom. Multi-DOFs, like hip or shoulder, are modeled with a series of one-DOF joints. More details about the used simulation software can be found in Vukobratović et al. (2007). The whole robot has 46 links and 52 DOFs since there are six additional DOFs for the free-flying base link. Kinematic structure of the robot is given in Figure 10.

The foot is not a single rigid object, but a two segment foot with a sole link and a fingers link. Both links were chosen in the form of trapezes and the contact between the foot and the ground is described by six points (four on the sole and two on the fingers). Each foot link was modeled as a rigid, nondeformable body with a thin, deformable visco-elastic layer of negligible mass. The visco-elastic layer was modeled as an isotropic Kelvin-Voigt material (Nikolić et al., 2014, 2018). Without loss of generality, all actuators were assumed to be the same, and modeled as DC motors with permanent magnets.

In order to verify the proposed bioinspired motion planning algorithm, simulation case studies have been conducted, demonstrating the robot's ability for online path planning and walk realization in an unstructured environment. In all simulations, the above-described model of humanoid robot was used.

### 3.1. Walking Around Walls

Simulation scenarios cover three different obstacle configurations, chosen to test the performance of the path planning algorithm. In simulation results, obstacles configuration and stick diagram of the robot motion are given. Additionally, footprints of the robot and projections of ZMP and CoM for the full path are plotted, which shows that dynamically balanced walk has been achieved, with successful obstacle avoidance. In each simulation, the goal of the robot is to reach a goal position given the coordinates x = 8 m and y = 0 m and the orientation *ϕ* = 0^*o*^.

**Figure 11:**
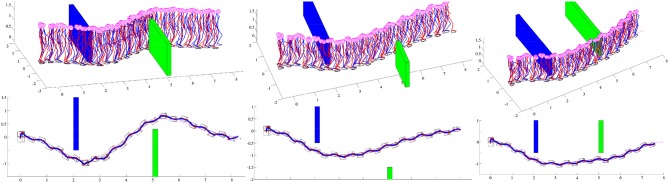
Walking in the environment with the different obstacle configurations: Stick diagram of robot motion **(top)** and robot footprints **(down)** with projections of ZMP (red cross) and PCM (blue circle).

In the first simulation experiment, the robot was supposed to walk from the starting point to the goal point in the environment which includes two parallel, nonplanar, overlapping walls. The results of the first simulation are shown in Figure 11 (left). The robot first tries to calculate the clothoid path directly from the starting point to the goal point and checks for the obstacles in the visible range. Since there is an obstacle in front of the robot (blue wall) robot finds an intermediate target point, in order to avoid the obstacle. A convex surface is calculated using three points, the robot's current position and two corners of the wall. Following the path planning algorithm, described in the previous chapter, two intermediate targets are calculated, since the obstacle may be circumvented from the left or from the right. The right target, which is closer to the shortest path without the obstacles, is chosen and the clothoid path is generated.

Each time when the robot starts new phase (see Figure 2), it recalculates a clothoid path, following the algorithm described in section 2.3. At this point a new obstacle (green wall) in front of the robot becomes visible. Since it is not possible to calculate a clothoid path directly to the goal point, because of the obstacle, the path planner calculates another path for obstacle avoidance. The path is calculated, which ensures the robot to bypass the green wall. When the robot avoids the second wall, the goal position becomes visible and the clothoid path toward the final goal position is generated.

The second simulation has a setup similar to the first one. There are also two parallel, nonplanar walls in the scene and the robot has to walk between the walls to reach the goal point. The major difference, compared to the first simulation scenario is that the robot, after circumventing the first wall has a clear sight to the goal position. Only one obstacle had to be avoided. The results of the second simulation are shown in Figure 11 (middle).

In the third simulation scenario, the robot is searching for the path in the scene with two parallel, non-planar walls. At the beginning of the simulation, the second wall (green one) is completely hidden behind the blue wall. Since there is an obstacle between the robot and the goal position, an intermediate target is calculated, to bypass the obstacle. Robot chooses the intermediate target on the right corner of the convex surface since it is closer to the robot's current position. After avoiding the first wall, another obstacle (green wall) is on the robot's path toward the goal position. Thus, a new convex surface is calculated and two intermediate targets, which fulfill the clearance condition are calculated. The closer target is chosen as an intermediate goal for the clothoid generation and the robot circumvents the second wall. When the robot avoids the second obstacle, the clothoid path which leads the robot to the goal point is found. The results of the third simulation are shown in Figure 11 (right).

### 3.2. Avoiding Moving Obstacle

For illustrating the possibility of the robot to plan the path online and adapt its walking parameters to follow the path, the following simulation scenarios are prepared. The robot's goal position is the same as in previous examples, i.e., x = 8 m, y = 0 m, *ϕ* = 0^*o*^. The obstacle, represented as a blue box that is moving from the right side of the robot, crosses the robot's path. The transparent blue trace represents the path of the obstacle. Same as is the section 3.1, the figure shows the stick diagram of the robot, the footprints and the projections of the ZMP and CoM of the robot.

**Figure 12:**
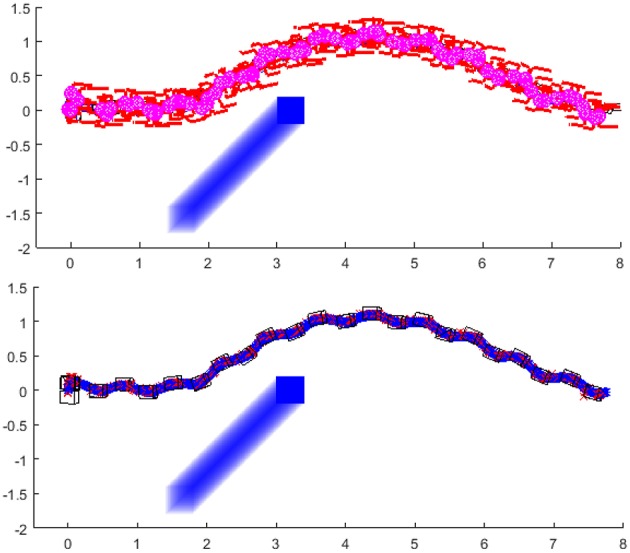
Robot is walking toward the goal position and turns left to avoid the obstacle: Stick diagram of robot motion **(top)**; footprints and projections of ZMP (red crosses) and PCM (blue circles) **(down)**.

The first example (Figure 12) shows the simulation of the robot walking toward the goal position, and the obstacle is crossing its path. The obstacle stops in front of the robot. The robot's path planning algorithm finds the point to generate the clothoid and turns left to avoid the obstacle. The obstacles stopped at the position in which the closest path to avoid the obstacle is always on the same side of the obstacle.

**Figure 13:**
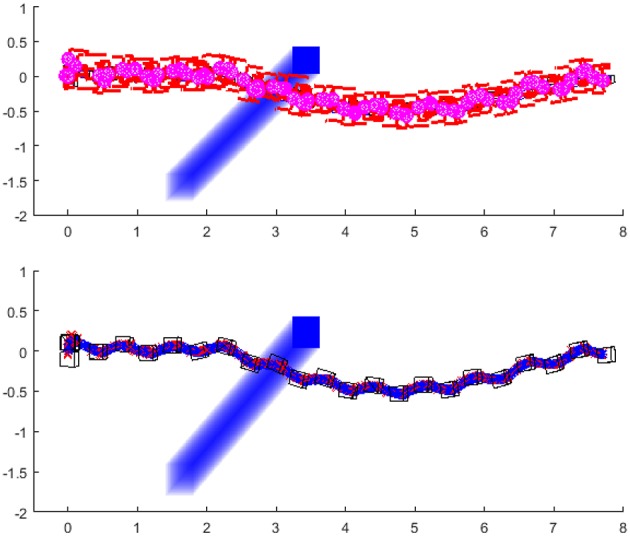
Robot first turns left and then turns right to avoid the obstacle with the shorter clothoid path around the obstacle toward the goal: Stick diagram of robot motion **(top)**; footprints and projections of ZMP (red crosses) and PCM (blue circles) **(down)**.

**Figure 14:**
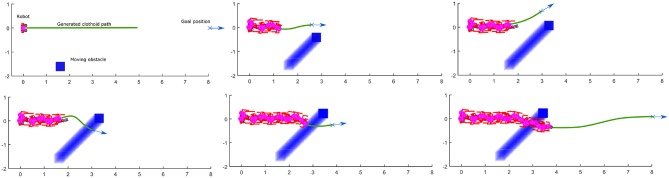
Sequence of figures illustrating the online path planing for obstacle avoidance.

The second example (Figure 13) shows the simulation of the robot walking toward the same goal position. The obstacle stops in front of the robot but in this case, the end position of the obstacle is such that the robot first finds the path to turn left, and then switches to turn right. The reason for this switch is that at one moment, the path to avoid the obstacle is shorter if the robot turns right. The sequence of the figure shown in Figure 14 illustrates the significant output of the path planning algorithm.

**Figure 15:**
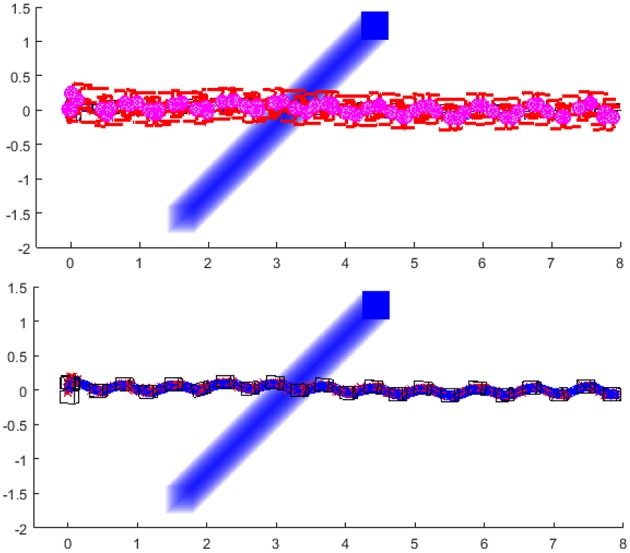
Robot slows down the walking speed, turns left and then turns right when the obstacle crosses the robot's path. After the obstacle is not crossing the path, the robot continues with normal speed toward the goal position following the shortest clothoid path: Stick diagram of robot motion **(top)**; footprints and projections of ZMP (red crosses) and PCM (blue circles) **(down)**.

The last example (Figure 15) shows the simulation of the robot when the moving obstacle crosses its path, but instead of stopping in front of the robot, it continues to move and eventually clears the way for the robot to pass. In this example, we introduced the rule to slow down the walking speed when the obstacle is crossing the robot's shortest path toward the goal. When the obstacle crosses the robot's path, the robot will slow down and plan to turn left in order to avoid the obstacle. When the obstacle is out of the robot's path, the robot calculates the new path toward the goal and speeds up to normal walking speed^4^. The link to the simulation experiment video of the robot avoiding the obstacles can be found in Supplementary Material.

## 4. Conclusion

This paper presented a novel methodology for biped walk path planning and walk synthesis based on motion primitives and neurological observations of human behavior. The presented approach showed the capacity for online modification of walk in an unknown environment. The first simulation showed how the robot can modify its walking parameters online, in order to avoid obstacles and walk between them. Results from Figure 11 highlighted the path planning algorithm that finds a transitory position, and illustrated how a walk is modified without upfront calculation of reference foot placements, joint trajectories, and its execution. Simulation examples given in Figures 12–15 showed the ability of a path planning algorithm to change the walking path online and adapt the high-level walking parameters. The framework for online path planning and walk realization showed the potential when the surrounding environment is unknown and dynamically changing.

The algorithm for walk synthesis was interfaced to the algorithm for biologically inspired, human-like path planning based on clothoid arcs. The algorithm for path planning prescribes an automatic change of parameters of walk based on the shape of a generated path. The outcome parameters of walk change over time and the realized walk changes simultaneously to comply with the laws derived from physiological studies and clinical research.

The proposed path planning approach, based on clothoid arcs smooth concatenation, was compared with another approach based on particle swarm optimization. It was shown that our approach provides a path with a smaller curvature, but a higher curvature derivative, i.e., sharper change of curvature, thus leading to the sharper change in walking direction, but higher possible walking speeds.

In future work, we plan to take into account the derivative of the path curvature, beside the curvature itself, as another cost function for optimization, in order to obtain smoother changes of the walking direction. Also, we plan to analyze how ascending and descending stairs can be integrated into our path planning algorithm in order to avoid obstacles on the stairs. The focus will be on the analysis of a human approaching the stairs with static and dynamic obstacles and transition of the human-like behavior onto the biped humanoid robot.

## Author Contributions

MR developed the idea and defined the relationship between the overall walking parameters and RAMP parameters. SS contributed to the implementation and adaptation of non-linear sliding mode control and disturbance estimator and the extraction of walking parameters from planned clothoid curves. JS-V contributed to the development of the biologically inspired path planning algorithm and in structuring the paper. MN improved the dynamic model to better simulate the contact between the foot and the ground and upgraded the existing software for multibody dynamic simulation. His contribution was also in preparing and conducting the simulations. BB contributed to the development of the idea of biped walking using RAMPs and in defining simulation scenarios. All the authors participated in writing and proofreading.

### Conflict of Interest Statement

The authors declare that the research was conducted in the absence of any commercial or financial relationships that could be construed as a potential conflict of interest.

## Footnotes

^1^Initial and goal position refer to both position (i.e., *x* and *y* coordinates) and orientation (i.e., rotation about *z*-axis denoted by φ) of the robot's base coordinate frame in the pelvis.

^2^The robot is modeled to have the distance between the left and right shoulder joint 36 cm. The clearance in the simulation examples in this papers is set to 30 cm and thus the margin between the robot and the obstacle is 12 cm, which is considered to be enough to avoid the collision with the obstacle.

^3^This range is set to 5 m in this simulation experiment and can be changed to meet different requirements.

^4^The video simulations of the robot avoiding the obstacle can be found on the following link: https://youtu.be/WBCesxnep0s, and the source code with the software for general modeling tool and the simulation examples from this paper can be found at https://github.com/rakovicm/Online_Planning_Walking_Unknown_Environment
